# Systematic discovery of unannotated genes in 11 yeast species using a database of orthologous genomic segments

**DOI:** 10.1186/1471-2164-12-377

**Published:** 2011-07-26

**Authors:** Seán S ÓhÉigeartaigh, David Armisén, Kevin P Byrne, Kenneth H Wolfe

**Affiliations:** 1 Smurfit Institute of Genetics, Trinity College Dublin, Dublin 2, Ireland

## Abstract

**Background:**

In standard BLAST searches, no information other than the sequences of the query and the database entries is considered. However, in situations where two genes from different species have only borderline similarity in a BLAST search, the discovery that the genes are located within a region of conserved gene order (synteny) can provide additional evidence that they are orthologs. Thus, for interpreting borderline search results, it would be useful to know whether the syntenic context of a database hit is similar to that of the query. This principle has often been used in investigations of particular genes or genomic regions, but to our knowledge it has never been implemented systematically.

**Results:**

We made use of the synteny information contained in the Yeast Gene Order Browser database for 11 yeast species to carry out a systematic search for protein-coding genes that were overlooked in the original annotations of one or more yeast genomes but which are syntenic with their orthologs. Such genes tend to have been overlooked because they are short, highly divergent, or contain introns. The key features of our software - called SearchDOGS - are that the database entries are classified into sets of genomic segments that are already known to be orthologous, and that very weak BLAST hits are retained for further analysis if their genomic location is similar to that of the query. Using SearchDOGS we identified 595 additional protein-coding genes among the 11 yeast species, including two new genes in *Saccharomyces cerevisiae*. We found additional genes for the mating pheromone a-factor in six species including *Kluyveromyces lactis*.

**Conclusions:**

SearchDOGS has proven highly successful for identifying overlooked genes in the yeast genomes. We anticipate that our approach can be adapted for study of further groups of species, such as bacterial genomes. More generally, the concept of doing sequence similarity searches against databases to which external information has been added may prove useful in other settings.

## Background

**Figure 1:**
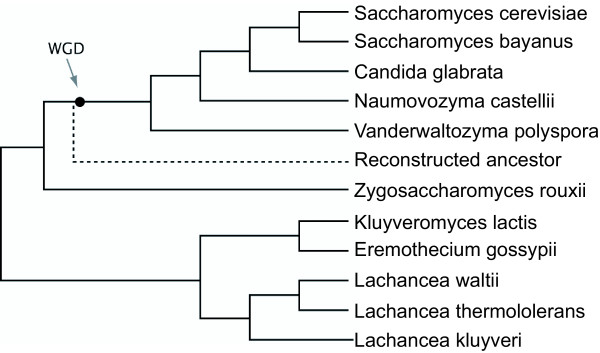
**Phylogenetic relationship among the 11 yeast species used in this study**. WGD indicates the position of the whole-genome duplication. The position of the inferred Ancestral genome [15] is indicated. Tree topology is from [44].

Yeast species have many features that make them an attractive model system for eukaryotic comparative genomics. These features include a high level of synteny conservation and small genome sizes (9-21 Mb) due to a low content of repetitive DNA and few introns [1,2]. We previously developed an online tool - the Yeast Gene Order Browser (YGOB) - for comparing local gene order relationships among species in genera such as *Saccharomyces*, *Kluyveromyces *and *Lachancea *[3]. YGOB now contains genomic data from 11 species (Figure 1). Among these species, some form a clade of descendants from an ancestral whole-genome duplication (WGD) that changed the basal chromosome number from 8 to 16 [4], whereas others diverged before the WGD occurred. We are unsure what depth of evolutionary time is represented by the species in YGOB, but when measured in terms of average protein sequence divergence this group of yeasts is approximately as diverse as the whole phylum Chordata [5].

YGOB contains 'pillars' of homology assignments across the 11 species. Each pillar can contain up to one gene from each non-WGD species and up to two genes from each post-WGD species [3]. The genes in a pillar are therefore orthologs or (in the case of a post-WGD species retaining two genes) paralogs resulting from the WGD. The pillars have undergone several years of manual editing to make them as accurate as possible. YGOB also contains an 'Ancestral Genome', which is the inferred gene content and gene order of the extinct ancestor that existed immediately prior to WGD [6].

The gene annotations in YGOB are derived from the original authors' annotations of the genome sequence of each species. In some cases we have 'switched off' genes in the original annotation that we believe to be spurious, but until now we have not added any genes to the original annotation sets (or to the current Saccharomyces Genome Database [7] annotation for *S*. *cerevisiae*). However, while using YGOB we noticed many instances in which a particular gene appears to be missing in a particular species, in a genomic region that otherwise shows conserved synteny among all the species. Such loci appear as gaps in the YGOB display. For the post-WGD species it is quite common for one of the two paralogs formed by WGD to have been deleted, but it is more surprising to find genes that are completely absent (zero copies) in either a non-WGD or a post-WGD species.

Upon further examination we found that many of these apparently zero-copy loci are artifacts. When we examine the relevant DNA region, we find *bona fide *genes that were not annotated or were mistakenly labeled as pseudogenes, even in the case of highly curated genomes. This is particularly a problem with short genes of less than 100 codons, highly diverged genes, and genes containing introns. In some cases, all genes <100 codons were excluded entirely from the original curators' annotations due to the difficulty in telling these apart from spurious ORFs [8,9]. However, current estimates according to the Saccharomyces Genome Database (SGD) [10] are that the *S*. *cerevisiae *nuclear genome contains 131 verified ORFs of <100 codons and even among these, 28 contain introns. Detecting these 'missing genes' is important for many reasons, but our particular interest in this topic is that it would allow the correct identification of genuine lineage-specific gene gains and losses which may have evolutionary significance.

The primary reason why short genes are difficult to annotate is that they do not generate sufficiently strong hits (low *E-*values) in BLAST searches [11]. For instance the amino acid sequence of ribosomal protein L41 is nearly identical among all the species in YGOB, but because this protein is only 25 residues long the BLASTP E-value between any two Rpl41 sequences is only of the order of e-07 to e-06. Many annotation pipelines would regard such a hit as insignificant, because *E-*values of this magnitude are often obtained purely by chance when longer query sequences are used. More generally, any gene whose predicted protein product cannot generate a significantly strong BLAST score against its orthologs will tend to remain unannotated. Weak BLAST scores can be caused by very rapid sequence divergence [12,13], or a high content of repetitive sequence that is masked by sequence-filter software [14], as well as by short sequence length.

In this manuscript we describe SearchDOGS, a piece of software that works in conjunction with BLAST [11] to identify unannotated genes. It is particularly designed to find genes that generate only weak BLAST hits, but whose syntenic context indicates that they are genuine orthologs to known genes. The major feature of SearchDOGS is that the genomes in the nucleotide sequence database used in the BLAST search have been pre-processed to subdivide them into sets of genomic regions that are already known to be orthologous. DOGS is an acronym for Database of Orthologous Genomic Segments. The BLAST results can then be post-processed to identify cases, even with very high *E-*values, where (i) the query protein hits genomic regions from multiple species in the database, and these regions are orthologous; or (ii) the syntenic context of the query protein is known, and it matches that of one or more of the database entries it hits.

SearchDOGS was initially developed as a standalone tool for displaying the syntenic contexts of the genomic hits obtained in a TBLASTN search using a single protein query. We then adapted it to carry out an automated and systematic search for unannotated genes in the genomes of all 11 yeast species in YGOB. Because the detection of a small or highly-diverged gene in one species may in turn make it possible to detect further orthologs of this gene in other species when the first gene is used as a query, we re-ran successive iterations of SearchDOGS on the yeast genomes until the program failed to find any more new genes.

## Results

### Orthologous genomic segments

**Figure 2:**
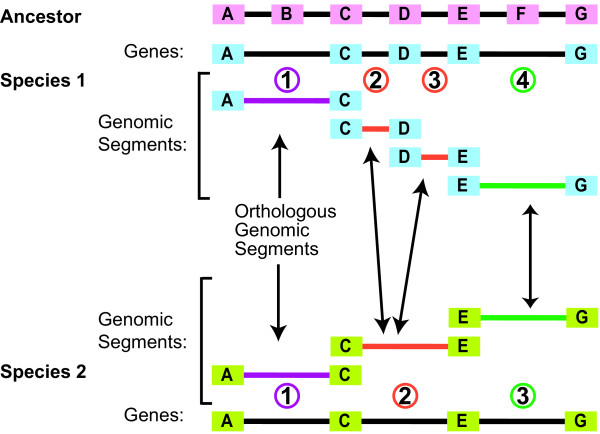
**Definition of orthologous genomic segments**. The genome sequences of species 1 and 2 are subdivided into overlapping regions, each containing two annotated genes and the intergenic DNA between them. The segments from species 1 and 2 in this example are classified into three orthologous genomic segment (OGS) groups, as indicated by coloring. Letters A-G represent genes in the Ancestral genome, some of which are retained in species 1 and 2.

The key concept behind SearchDOGS is that the nucleotide database that is searched by BLAST is organized into sets of sequences called Orthologous Genomic Segments (OGSs). We split up each of the 11 yeast genome sequences (Figure 1) used in YGOB into overlapping segments. Each segment consists of two adjacent annotated genes and the intergenic sequence between them (Figure 2). A BLAST nucleotide database ('DOGS') containing all the segments from all 11 species was then constructed. Separately, we mapped the two genes contained on each segment to the Ancestral yeast genome, which represents the gene order that existed just prior to the WGD event [15]. For each interval between two adjacent genes in the Ancestral genome, we were then able to identify genomic segments in the 11 modern species that are orthologous to this interval. A segment in a modern species can be orthologous to several consecutive intervals of the Ancestral genome due to gene deletions (Figure 2). Segments that are orthologous to the same Ancestral interval constitute an OGS group.

**Figure 3:**
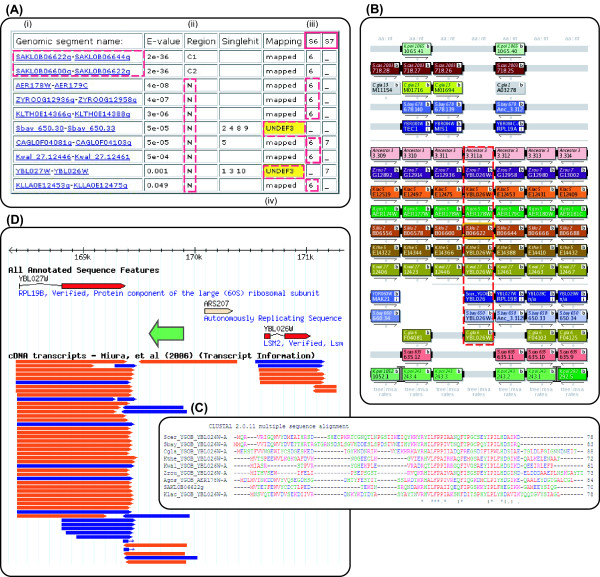
**Orthologs of the *L. kluyveri *gene *SAKL0B06622g *discovered in eight species**. (A) Output for web SearchDOGS with *SAKL0B06622g *used as a query. (i) The dashed box highlights the genomic segment containing the query. (ii) The letter N indicates the hits to noncoding regions in other species; C1 and C2 indicate coding regions. (iii) S6 and S7 refer to segments of the Ancestral genome. Genomic segments in other species that map to the same Ancestral segments constitute an OGS group. Six noncoding hits map to the same ancestral region (S6) as the query (dashed red boxes) and the seventh, located between *YBL027W *and *YBL026W *in *S*. *cerevisiae*, can be mapped to an adjacent ancestral region. (iv) 'UNDEF3' for the *S*. *bayanus *and *S*. *cerevisiae *genomic segments indicates that they have undergone some rearrangement relative to the Ancestor. The Ancestral segments to which they map are listed as "singlehit" if they are not shared with any other species. (B) YGOB screenshot after addition of the new genes, which are indicated by the dashed box. An ortholog of this gene is also inferred to have existed in the Ancestral genome because it is present in both non-WGD and post-WGD species. (C) ClustalW multiple sequence alignment [45] of the inferred protein sequences from nine species. (D) Location of the newly inferred *S*. *cerevisiae *gene *YBL026W-A *(green arrow), superimposed on a screenshot of the relevant region of chromosome II from SGD [46]. Eight expressed sequence tags from Miura *et al*. [16] indicate transcription of the gene.

SearchDOGS was initially developed as a standalone program with a web interface http://wolfe.gen.tcd.ie/searchDOGS, designed to search a single query protein against the DOGS database using TBLASTN (Figure 3A). Genomic segments hit in the search are identified in terms of their OGS groups. A typical protein query will hit the genomic segments that contain the annotated coding sequences of its orthologs in different species, which will constitute an OGS group. The BLAST HSPs (high-scoring pair alignments) of these hits will occur within the parts of the genomic segment that are already annotated as protein-coding, rather than the intergenic DNA between them. However, if in a particular species an ortholog of the query protein exists but has not been annotated, the DNA coding for it will have been annotated as intergenic DNA but the genomic segment containing this DNA will be part of the same OGS group as the orthologs in other species. For example, in Figure 2, if gene D exists in species 2 but has not been annotated, a TBLASTN search using gene D from another species as a query will hit an apparently noncoding region of segment 2 from species 2, as well as hitting coding regions of segments 2 and 3 from species 1. These three segments will all be in the same OGS group. So, by highlighting TBLASTN hits that occur in regions of database entries that are supposedly noncoding, we can identify possible unannotated orthologs of the query. We can consider even very weak hits between the query and noncoding regions of database entries, because we can reject any database hits that are not in the relevant OGS group.

As an example of results from the standalone SearchDOGS application, we found orthologs of the small (70 codons) *L*. *kluyveri *gene *SAKL0B06622g *in eight other yeast species in which it had not previously been annotated: *S*. *cerevisiae, S*. *bayanus, C*. *glabrata, Z*. *rouxii, K*. *lactis, A*. *gossypii*, *L*. *thermotolerans *and *L*. *waltii*, with *E *values ranging between 4e-08 and 0.049 (Figure 3A, B). In each of these noncoding regions an intact open reading frame was found, ranging in length from 61-88 codons, and showing significant amino acid sequence conservation (Figure 3C). When used as a BLASTP query the *S*. *cerevisiae *ORF, which we named *YBL026W-A*, hits *SAKL0B06622g *with an *E *value of 6e-04, and hits the other ORFs with *E *values ranging from 4e-21 to 0.009. These ORFs have been added to the YGOB database as new genes. Analysis of expressed sequence tag data [16] confirms expression of the newly identified *S*. *cerevisiae YBL026W-A *on the correct strand (Figure 3D). Prior to this study *SAKL0B06622g *appeared to be a species-specific gene in *L*. *kluyveri*, with no homologs annotated in the ten other YGOB species. These discoveries mean that orthologs of the gene are now known to exist, at a conserved location, in 9 of the 11 yeast species. We have not been able to find orthologs in the remaining two species (*V*. *polyspora, N*. *castellii)*.

### Automation and cycling

As we began to use the standalone SearchDOGS program, it became clear that due to the large number of hits and prospective genes being identified it would be necessary to automate the program to run over entire genomes. The automated version of SearchDOGS uses a modification of the original approach for increased speed and a slight increase in accuracy of synteny identification. The intergenic sequence between the two annotated genes in each genomic segment is used as a BLASTX query against a small database consisting only of the protein sequences of the genes that are syntenic with the query genomic segment. If a syntenic gene is hit, the region is retained for further processing. We retain all BLASTX hits with an *E-*value lower than 10, a very liberal cutoff.

We then test whether an intact gene structure with a protein sequence showing homology to the syntenic proteins can be identified within the intergenic region of the genomic segment. We use the program GetORF from the EMBOSS package [17] to generate a list of open reading frames located in the intergenic region. We use the protein translation of each ORF in the list as a BLASTP query against the syntenic YGOB pillar, and retain ORFs that hit the expected pillar. As well as this verification of synteny conservation we also require several other criteria to be met before an ORF is proposed as a genuine gene (Additional file 1: Figure S1), such as a comparison of the length of the HSP generated using the protein translation of the ORF as a BLASTP query against the syntenic pillar relative to the median length of the genes in that pillar. Finally, all proposed new genes are inspected by eye, considering their BLASTP results and a T-COFFEE multiple sequence alignment with other proteins in the pillar, for manual acceptance or rejection.

We ran a total of six cycles of the automated SearchDOGS program. In each cycle, genes discovered in the previous cycle were added to the query set. We also made modifications to the program between the cycles, to extend the range of situations it could deal with. The modifications included steps to automatically annotate intron-containing genes (see Methods), and modification of the synteny filter to allow unannotated genes to be detected in regions of genomes that have undergone rearrangement relative to other species. We also developed a method for dealing with pseudogenes. Pseudogenes are relatively rare in yeast genomes, but a few dozen have been described in *S*. *cerevisiae *and it is likely that similar numbers exist in other species [18,19]. In addition, there are many degenerated fragments of mobile elements such as Ty retroelements in yeast genomes. These pseudogenes are detected by SearchDOGS but it is not possible to annotate a corresponding intact gene. To prevent these loci being rediscovered in each cycle, we flagged them as pseudogene-containing regions and excluded them from the results of subsequent SearchDOGS runs.

### Automated SearchDOGS results

After six cycles of SearchDOGS we reached the point where no additional candidate genes were detected. The cumulative results of the six cycles are summarized in Table 1.

**Table 1:** New genes identified in 11 yeast species after six iterations of SearchDOGS.

		New genes added			Existing genes modified			Genes removed
		
Species	Updated number of genes	Total genes added	Intron- containing genes added	Frameshift/internal stop corrected	Total genes modified	Intron modified	Frameshift/internal stop corrected	
*V. polyspora*	5510	16	0	1	10	8	1	0
*N. castellii*	5688	18	1	1	13	9	1	1
*C. glabrata*	5224	16	0	1	6	3	2	1
*S. bayanus*	5223	258	142	3	17	8	7	3
*S. cerevisiae*	5606	2	0	1	2	0	2	1
*Z. rouxii*	5039	35	2	4	5	3	1	0
*K. lactis*	5120	40	4	9	4	2	1	1
*A. gossypii*	4742	17	2	0	3	0	1	0
*L. kluyveri*	5393	54	3	10	4	3	1	1
*L. thermotolerans*	5158	51	5	6	4	2	1	2
*L. waltii*	5275	88	56	1	38	19	13	4

Total		595	216	37	105	57	31	14

We added 595 new genes to the YGOB database, which can be viewed at http://wolfe.gen.tcd.ie/ygob (version 5: January 2011). A complete list of new genes is available at http://wolfe.gen.tcd.ie/searchDOGS. Of these, the largest proportion (43%) were in *S. bayanus*, which is still relatively poorly studied and annotated [13,20]. However, new genes were discovered in every species included. We were surprised to find two new genes in *S. cerevisiae *and 17 new genes in *A. gossypii*, given that these genomes have already been annotated to a very high standard [10,21]. The two new genes in *S. cerevisiae *are *YBL026W-A *(Figure 3) and *Scer_YGOB_Anc_7.495*. The latter gene, located between *YJR107W *and *YJR108W*, contains a frameshift in the 'reference' *S. cerevisiae *genome sequence of strain S288c, but not in alternative sequences of S288c obtained by Liti et al [22] and Miura et al [16], nor in sequences from other *S. cerevisiae *strains. In *A. gossypii *our results are supported by a recent resequencing and reannotation project that independently identified 15 of the 17 genes we discovered using SearchDOGS (Dietrich, F.S. et al, unpublished data. GenBank:AE016819, GenBank:AE016899-AE016904). During the course of the study we also identified a large number of genes across all species that were in need of modification or removal, due to errors such as a failure to annotate an intron, or partition of a single gene into multiple fragments due to frameshifts (Table 1). For some loci we found that a new gene could only be annotated in a particular species if apparent sequencing errors were overcome. We took a pragmatic approach to these loci: if a gene appeared to be intact except for one frameshift site or one internal stop codon, we annotated it and assumed that the problem was a sequencing error; if a gene contained more than one such site, we assumed that it is a pseudogene.

The list of new genes identified using SearchDOGS is heavily enriched for short genes: 64% of them are <200 codons long, and 38% are <100 codons. Most of them have yet to be assigned probable functions. By comparison, in the YGOB genome annotations of *S. cerevisiae *and *C. glabrata*, 16-17% of genes are <200 codons, and 3% are <100 codons. The large number of short genes discovered by SearchDOGS indicates not only that our approach is highly effective at detecting short genes, but also that a significant proportion of short genes have remained undiscovered to date.

For each new gene that we added, we calculated the ratio of nonsynonymous-to-synonymous nucleotide substitutions (*Ka/Ks*) between it and the other genes in the same YGOB pillar using PAML [23]. In all cases we found that the ratio was less than 1, indicating natural selection to preserve the amino acid sequence of the encoded protein.

### Examples of genes discovered by SearchDOGS

#### Highly divergent genes

The *S. cerevisiae *gene *NTC20*, coding for a protein required for pre-mRNA splicing, originally had orthologs annotated in all species except *A. gossypii*. SearchDOGS found a syntenic ortholog in *A. gossypii*, but the protein sequence divergence between it and the *S. cerevisiae *ortholog is so large that they do not hit one another in a BLASTP search (*E *> 10), which is probably the reason that the gene was overlooked in the original *A. gossypii *annotation [24] even though it is relatively long (171 codons). SearchDOGS initially found a hit to this genomic region in *A. gossypii *by using the *Z. rouxii *ortholog (*ZYRO0A13266g*) as a BLAST query. The *A. gossypii *ORF is confirmed as an *NTC20 *ortholog because it hits six other proteins from the *NTC20 *YGOB pillar when used as a BLASTP query. All six of these hits have very high *E-*values (ranging from 0.11 to 8.5), and the other four proteins in the pillar must have *E-*values greater than 10. *NTC20 *is an exceptionally divergent gene: none of the 11 *NTC20 *orthologs in the YGOB pillar is able to detect all the other members of the pillar with a BLASTP *E-*value below 10.

We also found new orthologs of *S. cerevisiae REC107*, an intron-containing gene that is involved in the early stages of meiotic recombination. At the start of this study orthologs were only known in the other post-WGD species and in *A. gossypii*. SearchDOGS identified divergent orthologs of *REC107 *in all the other non-WGD species (*Z. rouxii, K. lactis, L. kluyveri, L. thermotolerans, L. waltii*), with BLASTP *E-*values to the *S. cerevisiae *protein ranging from 5e-19 to 6e-11.

#### Genes for a-factor

*MFA *genes coding for the **a**-factor mating pheromone in budding yeasts are known to be difficult to identify due to their short size (32-38 residues), high sequence divergence and an apparently high rate of gene duplication or transposition [25]. Using a combination of SearchDOGS and standard TBLASTN searches we identified ten unannotated *MFA *genes: three in *N. castellii*, two in *K. lactis *and *L. kluyveri*, and one each in *Z. rouxii, L. waltii*, and *L. thermotolerans*. A previous study by Ongay-Larios *et al*. [26] identified and knocked out one of the *K. lactis MFA *genes but did not notice the second gene.

**Figure 4:**
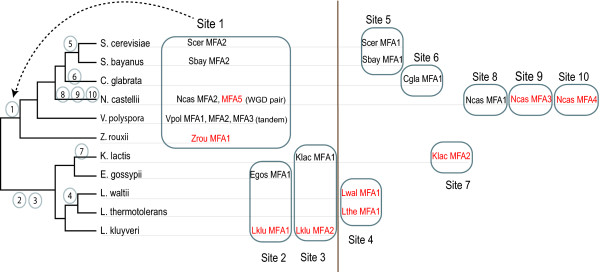
**Summary of *MFA *(a-factor) gene locations in 11 yeast species**. Sites 1-10 indicate the ten different genomic locations at which *MFA *genes are found. *MFA *genes newly discovered by SearchDOGS are highlighted in red. Numbers on the phylogenetic tree indicate the earliest branches to which each location maps. Sites to the right of the vertical line indicate recent species- or genus-specific gene movements.

An analysis of *MFA *gene locations reveals a complex history of gene duplication and relocation (Figure 4). For example *N. castellii *has five *MFA *genes, two of which are a pair formed by WGD and located at a site that is conserved with most other post-WGD species and *Z. rouxii *(Site 1 in Figure 4), but the other three *N. castellii *genes are at locations that are not shared with any other species. Among the 11 species, all but three of the ten separate genomic sites where *MFA *genes are currently located represent new sites to which *MFA *moved in the time since the WGD occurred (Figure 4).

#### Discovery of new divergent ohnolog pairs in S. cerevisiae

In three instances, the discovery of orthologs of *S. cerevisiae *genes in non-WGD species led us to realize that a pair of *S. cerevisiae *genes are ohnologs (paralogs produced by the WGD). The first pair is the *S. cerevisiae *genes *HOR7 *and *DDR2 *(59 and 61 codons, respectively). We initially discovered that *HOR7 *has unannotated syntenic orthologs in the non-WGD species *K. lactis, L. kluyveri *and *L. waltii*, and then found that these non-WGD genes were also syntenic with, and had weak similarity to, *S. cerevisiae DDR2*. There is no direct BLASTP hit between the two *S. cerevisiae *proteins. The second pair is similar: *S. cerevisiae YDR524C-B *(66 codons) and *YCL048W-A *(79 codons) were found to be an ohnolog pair, because they are both syntenic with, and have weak similarity to, a newly discovered gene in each of *L. kluyveri*, *L. thermotolerans *and *L. waltii*. PSI-BLAST searches show that these four small *S. cerevisiae *proteins are members of a single family, but their precise function is ill-defined. *HOR7 *and *DDR2 *are known to be upregulated in response to stress. *HOR7 *is responsive to hyperosmolarity [27] and *DDR2 *is a member of a family of multistress-responsive genes [28]. Both *HOR7 *and *YDR524C-B *are expressed across a range of conditions, although whereas *HOR7 *is upregulated in response to heat shock, *YDR524C-B *is downregulated. Both *DDR2 *and *YCL048W-A *have low expression in rich media but are upregulated in response to ethanol or heat shock [29,30].

**Figure 5:**
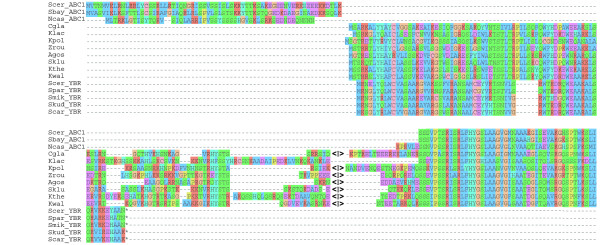
**Alignment of Abc1, Ybr230w-a and orthologous proteins**. Only the N-terminal region of Abc1 is shown, and the positions of introns are marked by <I>. The alignment was made using MUSCLE as implemented in Seaview [47]. The *YBR230W-A *genes in the *Saccharomyces *species (*S. cerevisiae, S. paradoxus, S. mikatae, S. kudriavzevii *and *S. carlsbergenesis) *align to the first exon of the two-exon *ABC1 *gene in *V. polyspora, Z. rouxii, A. gossypii, L. kluyveri, L. thermotolerans *and *L. waltii *whereas *ABC1 *of *S. cerevisiae, S. bayanus *and *N. castellii *align only to the second exon.

The third pair of newly-recognized ohnologs are *S. cerevisiae ABC1 *and *YBR230W-A *[31], a small gene previously identified by McCutcheon and Eddy [31]. Search-DOGS identified that *YBR230W-A *(which was originally 'switched off' in YGOB's *S. cerevisiae *annotation) and *ABC1 *hit the same genes in non-WGD species. *ABC1 *and *YBR230W-A *have an unusual history because they no longer retain any homologous sequence. After WGD, the two ohnologs retained only different, non-overlapping parts of the original gene. Consequently, *ABC1 *and *YBR230W-A *cannot be aligned to one another, but they both align to parts of a longer gene in non-WGD species that is orthologous to both of them (Figure 5). *S. cerevisiae ABC1 *(501 codons) is a large single-exon gene that corresponds to exon 2 of its orthologs in non-WGD species. *YBR230W-A *(66 codons) shows high similarity to exon 1 of the gene in non-WGD species (Figure 5), and is conserved within the genus *Saccharomyces *[31]. It appears that after WGD, one *S. cerevisiae *gene *(ABC1) *lost exon 1 and the other *(YBR230W-A) *lost exon 2 in a reciprocal fashion. Thus, these two genes that show no sequence similarity to each other share a common ancestor.

The origin of *ABC1 *and *YBR230W-A *by fission of an ancestral gene raises a puzzle about the origin of *ABC1*'s mitochondrial import signal. *S. cerevisiae *Abc1 is a mitochondrial protein that is involved in activation of the cytochrome bc1 complex and is required for coenzyme Q biosynthesis [32-34]. It is imported into the mitochondrion by means of a signal sequence at its amino terminus. Ybr230w-a and the proteins from non-WGD species are also predicted bioinformatically to be targeted to mitochondria [35-38]. Since *ABC1 *did not retain the 5' end of the ancestral gene, it must have gained a new signal sequence upstream of the former exon 2. It is interesting to note that the *N. castellii *ortholog of *ABC1 *also appears to have lost exon 1, but there is no evidence that exon 1 exists in the form of a separate gene in that species. The function of *YBR230W-A *is unknown, but transcriptome data indicates that both *ABC1 *and *YBR230W-A *are expressed [29], and that expression of *YBR230W-A *is upregulated under heatshock conditions [30].

## Discussion

The principle underlying SearchDOGS is one that is familiar and intuitive - that orthologous genes should be located in orthologous genomic regions. For the yeast species considered here, this principle turns out to be useful for gene discovery, because their genomes have undergone relatively little gene order change while accumulating extensive gene sequence divergence [39]. The idea that two orthologous genes can diverge so much in sequence that they fail to hit each other in a BLAST search is somewhat unsettling, and we were surprised when we encountered the first examples of this phenomenon [12]. We can now quantify the phenomenon more precisely as follows. In our YGOB database there are 5108 pillars that contain at least two genes. Among these, 135 pillars (2.6%) contain at least two genes that do not hit each other at all, despite being orthologous (BLASTP search, *E *> 10, Blosum62 matrix, merging hits seen both with and without the SEG low-complexity filter). The orthology of these genes has been confirmed via hits to a third sequence in the same pillar, or via longer chains of hits [40].

Most annotation pipelines will only annotate a putative gene if it has significant similarity to another gene in a database, or if it has an ORF above a certain length threshold. Therefore it is not surprising that short, intron-containing, and highly-divergent genes tend to have been overlooked by the annotation process. SearchDOGS provides a method for finding these genes. In the near future it will probably also become possible to detect them using high-throughput transcription data such as RNA-seq [41], but at the moment we have many genome sequences from species whose transcriptomes remain unstudied. Also, RNA-seq data establishes that a locus is transcribed, but does not identify its orthologs in other species.

Although the principle behind our method is simple, to our knowledge SearchDOGS is the first attempt to apply this principle in a systematic and automated way. As well as the obvious advantage of speed, the automated approach has the additional advantage of robustness because it often finds multiple lines of evidence for the existence of the same gene. For example a *Z. rouxii *ortholog of *YPR036W-A *was detected in the intergenic region between *ZYRO0G17248g *and *ZYRO0G17270g *when it hit the *S. cerevisiae *protein in a search, but the same intergenic region also hit the orthologs of *YPR036W-A *from *V. polyspora, N. castellii*, and *A. gossypii*. In this way, searches using different members of the same pillar can back each other up, lending confidence to the predictions.

The only substantial problem we encountered using SearchDOGS was the difficulty of differentiating pseudogenes from unannotated but genuine genes. This is a particular problem for sequences that contain large ORFs but are nevertheless truncated relative to their orthologs in other species. Without experimental verification it will be difficult to distinguish between a functional gene that is shorter than its orthologs and a truncated pseudogene. Furthermore, the sequenced strain of a species may contain null alleles at some loci. These are loci at which the population is polymorphic with a mixture of functional and nonfunctional alleles. For instance, the *CRS5 *gene contains an in-frame stop codon in *S. cerevisiae *strain S288c but not in other strains [42]. Without information from other strains it is impossible to distinguish between a null allele and a pseudogene that is fixed in the population.

One limitation of SearchDOGS is that, to find an unannotated gene, it must use a gene currently annotated in another species as a query. Therefore it cannot find completely novel genes that are not annotated in any species. We tried to overcome this limitation by using TBLASTX searches (six-frame translations of a query DNA sequence compared to six-frame translations of the database) after the six cycles of automated TBLASTN/BLASTX searches were finished. However, this approach generated a very large number of spurious hits (attributable to translations of sequences such as retrotransposon LTRs and RNA-coding genes), and we did not find any genuine additional genes in the 11 yeast species using it.

## Conclusions

We have successfully used SearchDOGS to identify a large number of genes previously overlooked in the genomes included in YGOB. The principle of using local gene order information to inform searches for unannotated genes is completely generic so in principle the SearchDOGS method could be applied to many groups of organisms, although in its current implementation - without sophisticated gene structure modeling - it is best suited to species with few or no introns. The broad requirements for a SearchDOGS approach to be viable are as follows:

(i) The species must already be reasonably well annotated. SearchDOGS will find missing genes, but if the majority of genes in a species are missing SearchDOGS will have difficulty pinpointing the locations of new genes relative to those already identified.

(ii) The species in the dataset must not be too rearranged. SearchDOGS can only make predictions in regions of the genome where it can establish local synteny relationships.

(iii) A pillar structure (*i.e*., homology assignments for the genes) must exist or be generated. In our implementation we classified the genomic segments from each yeast species into orthologous groups (OGSs) by mapping them onto an Ancestral yeast gene order that we had previously determined [15]. For SearchDOGS to be applied to other systems, the user would need to nominate one genome as a reference onto which the OGS groups would be mapped.

Based on these requirements, we anticipate that SearchDOGS may prove useful in the future for finding unannotated genes in bacterial genomes, but it may be less useful in genomes with many introns and large noncoding regions, such as mammals, or in species that lack close relatives with well-annotated genomes.

## Methods

### Database and search method

The DOGS database was constructed using the genome sequence and gene annotations in the YGOB browser, data release 4 (May 2010) [3], which includes 11 species (Table 2). The Ancestral yeast gene order is from Gordon *et al*. [6]. For the standalone version of SearchDOGS we constructed a single nucleotide database containing all the genomic segments. This database can be searched using either TBLASTN or TBLASTX. For the early automated cycles of the program, the amino acid sequence of each protein in the YGOB database was used as a TBLASTN query against syntenic genomic segments. To reduce computation time we constructed a specific small database for use with each pillar's queries, containing only the genomic segments that are syntenic to it. The TBLASTN searches used cutoffs of *E *< 10 and 100 results listed, with the low-complexity SEG filter turned off. Hits to noncoding regions syntenic with the query protein were retained.

**Table 2:** Genome sequences and annotations used in the SearchDOGS database.

Species	Coverage	Sequence	Gene annotation
*V. polyspora*	7.8x	[48]	[48]
*N. castellii*	4x	[20]	Wolfe laboratory, based on Cliften et al. [20]
*C. glabrata*	Complete	[49]	Dujon et al. [49]
*S. bayanus*	6.4x	[13]	[13]
*S. cerevisiae*	Complete	[8]	Wolfe laboratory, based on SGD 2009 release
*Z. rouxii*	Complete	[50]	[50]
*K. lactis*	Complete	[49]	Dujon et al. [49]
*A. gossypii*	Complete	[51]	Dietrich et al. [51]
*L. kluyveri*	Complete	[50]	[50]
*L. thermotolerans*	Complete	[50]	[50]
*L. waltii*	8x	[52]	Kellis et al. [52]

As subsequent iterations of SearchDOGS were run, modifications were made to improve the initial synteny-determining method, and to improve speed by using BLASTX instead of TBLASTN. The final method for establishing synteny is as follows: For each genomic segment the pillars containing the flanking genes are retrieved. This information is used to map the intergenic region against the other species in YGOB. If no rearrangement has occurred between a given species and the species of the query, all the genes in that species between the flanking pillars are retrieved, making up a database against which the intergenic region of the genomic segment is searched (BLASTX) (Additional file 1: Figure S2). Otherwise we 'step out' from one flanking pillar towards the other, retaining each gene until we reach a gene for which pillar information shows that synteny with the intergenic sequence has been lost, or up to a maximum of 10 genes from the pillar (Additional file 1: Figure S2).

### Intron and frameshift prediction

Open reading frames within the regions of interest identified by SearchDOGS are obtained using GetORF with default parameters [17] except that the minimum ORF size is 60 nucleotides (start to stop). The set of ORFs generated by GetORF are subjected to a first step of analysis using BLASTP, as described in the Results. This step identifies coding regions that are free of frameshifts and consist of a single exon (Additional file 1: Figure S1). Next, results are subjected to a second step of analysis designed to identify genes containing frameshifts or introns. In this step we look for pillars of genes that map to the intergenic region of the genomic fragment in which a BLAST hit has been found. If a potential ORF within the intergenic region contains a single frameshift that can be corrected to translate to a protein similar to other genes in the homologous pillar, it is considered real and is corrected. The location of the frameshift is an estimate, and therefore the ORF is flagged for manual verification.

We anticipate that a newly discovered gene might contain an intron if one or more of the genes in the YGOB pillar that hits it contains an annotated intron. In the case of pillars of genes containing introns, TBLASTN is used to search the protein sequence of each of the exons of the genes in these pillars against the intergenic region of the fragment to define potential exons within the fragment. If two or more potential ORFs have the same order as the exons of any genes in the syntenic pillar, and if the lengths of the ORFs are within 10 amino acids of the lengths of the exons in the pillar, an intron is predicted and we search for splice sites (GT-AG) associated with the boundaries of the intron. No frameshifts are allowed when an intron is predicted. If the TBLASTN hits do not include start and stop codons, an enlargement of the coding region of up to 40 amino acids is allowed until start and stop codons are reached. The final protein length is tested against the median protein length of the homologous pillar for a measure of prediction confidence. Exons smaller than 20 codons are difficult to identify by BLAST, so if a pillar that generates a hit contains a small exon, only the larger exon(s) are usually detected in a TBLASTN search, and therefore the hit is flagged for manual annotation.

New genes identified using SearchDOGS were added to the YGOB database and given temporary names containing the tag 'YGOB' such as *Zrou_YGOB_Anc_5.606 *to indicate a *Z. rouxii *ortholog of the gene at ancestral position Anc_5.606 [15]. We will communicate lists of these loci to the relevant databases so that permanent names can be assigned to them.

### Criteria for rejection of hits

A BLASTX hit between a genomic segment and a protein from an orthologous YGOB pillar could be rejected, either automatically (Additional file 1: Figure S1) or after manual inspection. The most common reasons why hits between a genomic segment and an orthologous protein were rejected were:

- Segments did not contain an intact ORF, due to multiple stop codons and/or frameshifts. These were classed as pseudogenes.

- BLAST relationship was not reciprocal: the intergenic sequence of a genomic segment had a BLASTX hit to a protein in an orthologous YGOB pillar, but when the translated ORF from the genomic segment is used as a BLASTP query it failed to hit any of the proteins in the same pillar.

- The length of the HSP generated by the BLASTP search was not sufficiently long compared to the median length of the genes in the corresponding YGOB pillar.

- The translated ORF showed too little sequence similarity to existing genes in the pillar in a subjective inspection of a T-coffee alignment [43], and were therefore considered unlikely to be real.

- Segments syntenic to intron-containing pillars, for which we were unable to construct a convincing gene model.

## Authors' contributions

SOH carried out some of the software design and gene annotation, performed the statistical analysis and drafted the manuscript. DA carried out some of the software design and gene annotation. KB participated in the design of the study and provided technical advice. KW conceived of the study, and participated in its design and coordination and helped to draft the manuscript. All authors read and approved the final manuscript.

## Author information

Seán S. ÓhÉigeartaigh: PhD student, Wolfe Bioinformatics Laboratory. Dr. David Armisén: Postdoctoral researcher, Wolfe Bioinformatics Laboratory.

Dr. Kevin P. Byrne: Postdoctoral researcher, Wolfe Bioinformatics Laboratory

Professor Kenneth H. Wolfe: Principal Investigator, Wolfe Bioinformatics Laboratory; Professor of Genome Evolution, Trinity College Dublin.

## Supplementary Material

Additional File  1 **Supplementary material**. Two additional figures (Figures S1, S2). Click here for file
