# Supplementary material for: Systematic discovery of unannotated genes in 11 yeast species using a database of orthologous genomic segments
Source: BMC Genomics. 2011 Jul 26;12:377. doi: 10.1186/1471-2164-12-377 (PMC3161974; doi:10.1186/1471-2164-12-377)
Supplement: Additional File 1 — Supplementary material. Two additional figures (Figures S1, S2). [file 1471-2164-12-377-S1.DOC]

**Additional File 1: Supplementary figures and table.**

*
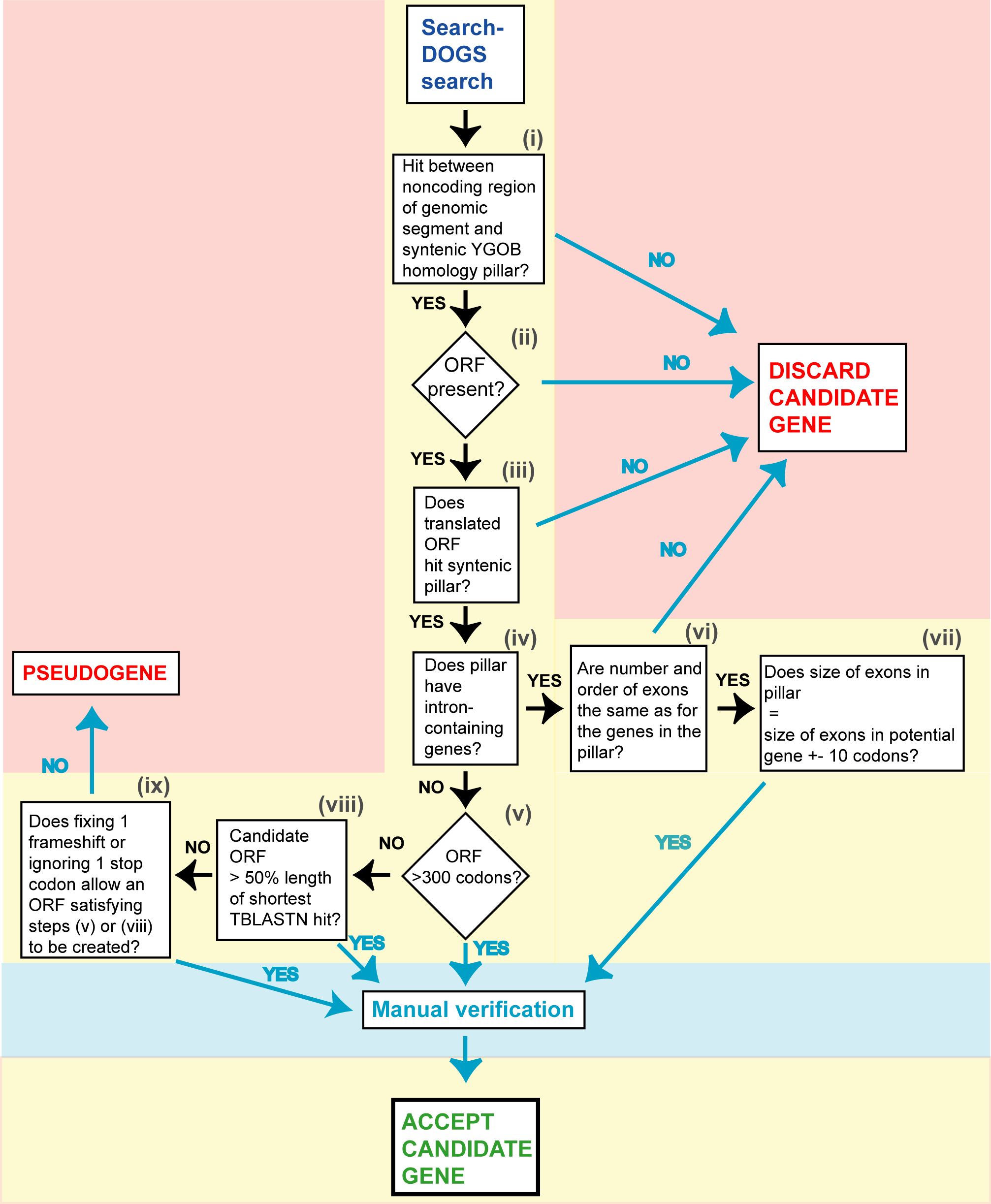
*

Figure S1: Flowchart illustrating the criteria that must be achieved for a SearchDOGS hit to be considered a *bona fide* gene. The automated steps are marked with roman numerals. All candidate genes that pass the automated steps are subjected to a manual examination before they are accepted.


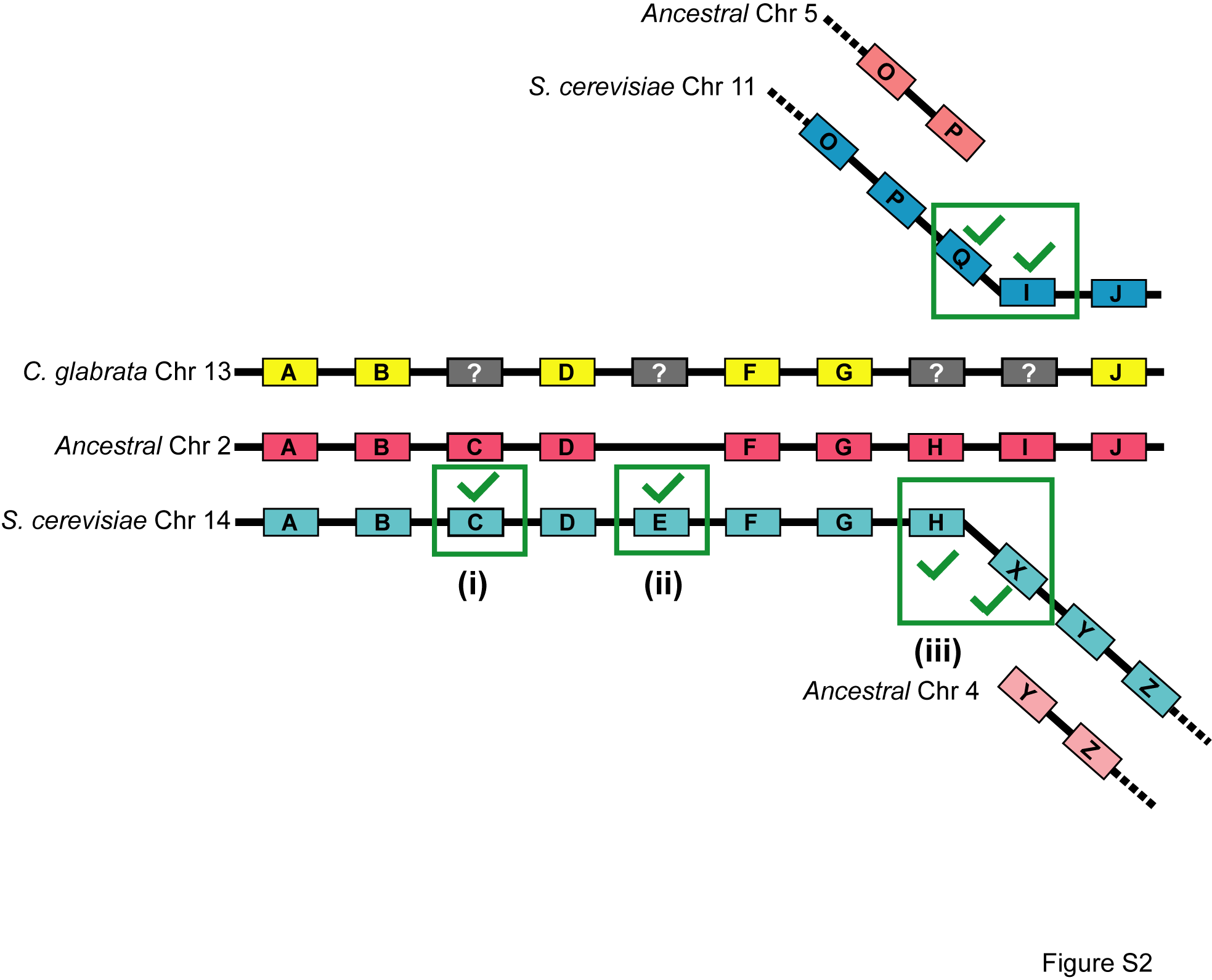


Figure S2: Cartoon illustrating the automated SearchDOGS method for establishing orthology between genomic segments. For ease of explanation, only two extant species are shown (*S. cerevisiae* in blue, and *C. glabrata* in yellow), as well as the Ancestral genome (pink; parts of Ancestral chromosomes 2, 4 and 5 are shown).

(i) Testing for a possible *C. glabrata* ortholog of gene *C*, which is included in the Ancestral genome. For the *C. glabrata* genomic fragment *B-D*, the two annotated genes have Ancestral orthologs (*Anc_2.B* and *Anc_2.D*) that are less than 10 ancestral genes apart. In this situation, we use the intergenic region between *C. glabrata B* and *D* as a BLASTX query against a database that contains the translations of all genes that map between *Anc_2.B* and *Anc_2.D.* Therefore *S. cerevisiae* gene *C* is included in this database (green tick and box).

(ii) Testing for a possible *C. glabrata* ortholog of gene *E*, which is not included in the Ancestral genome*.* For the *C. glabrata* genomic fragment *D-F*, the two annotated genes have orthologs in another species (*S. cerevisiae*) that are less than 10 genes apart. In this situation, we put each of the genes from that species (*i.e., S. cerevisiae* gene *E*) in the database against which the *C. glabrata* intergenic region will be searched using BLASTX.

(iii) Interspecies rearrangements. In this example, an interspecies rearrangement has occurred in *S.* *cerevisiae* relative to the Ancestor and *C. glabrata*, creating two new gene orders *G-H-X-Y-Z* and *O-P-Q-I-J* in *S. cerevisiae*. To search for possible unannotated genes in the interval between *C. glabrata* *G* and *J*, we define two orthologous *S. cerevisiae* genomic segments as follows. First, we consider the gene on the left end of the *C. glabrata* segment, *Cgla G*. We identify its *S. cerevisiae* ortholog from the same pillar (*Scer G*), and walk rightwards from this gene until we reach the point where synteny is lost (*Scer* *Y*; we know that synteny is lost because it is in a pillar with a different part of the ancestral genome, *Anc_4.Y*). We therefore put *S. cerevisiae* genes encountered on this walk (*Scer H* and *X*) into the database against which the *C. glabrata* *G-J* intergenic interval will be searched by BLASTX. Second, we similarly consider the gene on the right end of the *C. glabrata* segment, *Cgla J*, find its *S. cerevisiae* ortholog (*Scer J*) and walk leftwards in *S. cerevisiae* until synteny is known to be lost (at *Scer P*). We add the *S. cerevisiae* genes from this encountered on this walk (*Scer I* and *Q*) to the database. Thus the *C. glabrata* *G-J* intergenic region will be used as a BLASTX query against a database containing *S. cerevisiae* *H*, *X*, *Q* and I*.*
